# Phenol-Rich Food Acceptability: The Influence of Variations in Sweetness Optima and Sensory-Liking Patterns

**DOI:** 10.3390/nu13030866

**Published:** 2021-03-06

**Authors:** Sara Spinelli, John Prescott, Lapo Pierguidi, Caterina Dinnella, Elena Arena, Ada Braghieri, Rossella Di Monaco, Tullia Gallina Toschi, Isabella Endrizzi, Cristina Proserpio, Luisa Torri, Erminio Monteleone

**Affiliations:** 1 Department of Agricultural, Food, Environment and Forestry (DAGRI), University of Florence, via Donizetti 6, 50144 Florence, Italy; prescott18@gmail.com (J.P.); lapo.pierguidi@unifi.it (L.P.); dinnella@unifi.it (C.D.); 2 TasteMatters Research & Consulting, P.O. Box Q1150, QVB Post Office, Sydney 1230, Australia; 3 Dipartimento di Agricoltura, Alimentazione e Ambiente (Di3A), University of Catania, Via Santa Sofia 100, 95123 Catania, Italy; earena@unict.it; 4 School of Agricultural, Forest, Food, and Environmental Sciences, University of Basilicata, Via dell’Ateneo Lucano 10, 85100 Potenza, Italy; ada.braghieri@unibas.it; 5 Department of Agricultural Sciences, University of Naples Federico II, 80055 Portici, Italy; dimonaco@unina.it; 6 Department of Agricultural and Food Sciences (DISTAL), Alma Mater Studiorum—University of Bologna, 40126 Bologna, Italy; tullia.gallinatoschi@unibo.it; 7 Department of Food Quality and Nutrition, Research and Innovation Centre, Fondazione Edmund Mach (FEM), Via E. Mach 1, 38010 San Michele all’Adige, Italy; isabella.endrizzi@fmach.it; 8 Department of Food, Environmental and Nutritional Sciences (DeFENS), University of Milan, 20133 Milan, Italy; Cristina.proserpio@unimi.it; 9 University of Gastronomic Sciences, Piazza Vittorio Emanuele II, 9, 12042 Pollenzo, Italy; l.torri@unisg.it

**Keywords:** phenol-rich foods, sweet liking, sensory-liking patterns, individual differences, personality traits, PROP

## Abstract

The consumption of phenol-rich foods is limited by their prominent bitterness and astringency. This issue has been addressed by adding sweet tastes, which suppress bitterness, but this is not a complete solution since individuals also differ in their preference for sweetness. In this study, we aimed at identifying groups of consumers differing in sweetness optima and sensory-liking patterns. To this end, increasing concentrations of sucrose were added to a chocolate pudding base. This allowed us to (1) investigate if individual differences in sensory responses are associated with different sweet liking optima in a product context, (2) define the psychological and oro-sensory profile of sweet liker phenotypes derived using a product context, and (3) assess if individuals differing in sweet liking optima differ also in consumption and liking of phenol-rich foods and beverages as a function of their sensory properties (e.g., sweeter vs. more bitter and astringent products). Individuals (1208; 58.4% women, 18–69 years) were characterised for demographics, responsiveness to 6-n-propylthiouracil (PROP), personality traits and attitudes toward foods. Three clusters were identified based on correlations between sensory responses (sweetness, bitterness and astringency) and liking of the samples: liking was positively related to sweetness and negatively to bitterness and astringency in High and Moderate Sweet Likers, and the opposite in Inverted U-Shaped. Differences between clusters were found in age, gender and personality. Furthermore, the Inverted-U Shaped cluster was found to have overall healthier food behaviours and preferences, with higher liking and consumption of phenol-rich vegetables and beverages without added sugar. These findings point out the importance of identifying the individual sensory-liking patterns in order to develop more effective strategies to promote the acceptability of healthy phenol-rich foods.

## 1. Introduction

Bitter and astringent foods are unacceptable for a substantial proportion of the population. As a result, a large number of potentially healthy foods—those that are phenol-rich, for example such as some vegetables—face barriers to their consumption because of their prominent bitterness and astringency. A variety of strategies for moderating the bitterness of foods continue to be assessed, but the easiest approach is to add sources of sweetness or saltiness to foods, as both of these tastes are bitter suppressors [1,2,3]. From a health perspective, however, such an approach has obvious limitations, due to the modification of the nutritional content of the product.

Furthermore, adopting any overall strategy to suppress bitterness may be problematic because of the fact that consumers are not uniform in their perception of, or preferences for, bitterness, or to the tastes that modify bitterness. The most well-researched sensory segmentation of consumers is based on responses to the bitter compound 6-n-propylthiouracil (PROP). Responses to this compound, which are at least partly genetically determined [4], also reflect responses to other bitter compounds, as well as sweetness, sourness, and a variety of oral and odour irritant and textural attributes, in both solutions and foods/beverages [5,6,7,8,9,10,11,12,13,14,15].

Consumer segments based on sweetness liking have also been identified. While it has been suggested that there may be a universal optimum sweetness level [16,17], it has been well demonstrated that such average values are made up of multiple groups displaying differing sweetness optima [18,19,20,21]. These groups have sometimes been defined in terms of sweet likers vs. sweet dislikers, but up to five groups varying in optimal sweetness in solution across a range of sucrose concentration are shown in most studies, although three groups are most commonly identified based on their hedonic pattern: increased liking (“sweet likers”), increased disliking (“sweet dislikers”), and increasing liking ratings followed by a reduction for solutions with added sucrose above a specific concentration (“inverted U-shaped”) [18,20].

The extent to which consumer segments based on specific tastes are maintained for real foods or beverages is unclear. Those who are insensitive to PROP (non-tasters) tend to consume more sugars and fat but are also more likely to consume bitter vegetables [22,23,24,25], suggesting that those compounds that cause bitter tastes for others are acceptable. PROP non-tasters have also been found to develop preferences to flavours in which the odour component is paired with bitter-sweet saccharin, while PROP supertasters and, to a lesser extent, medium tasters do not [26]. However, results are mixed and other studies found no or limited effect of PROP status on food acceptability and intake of vegetables [27,28,29,30], encouraging further investigations.

The generalisation of classifications based on responses to sucrose solutions to sweet foods or beverages has also been investigated, with mixed results. Kim et al. [18] found three clusters of sweet liking based on ratings of both sweet solutions and beverages. Cluster 1 showed increasing positive hedonic ratings with increased sucrose concentration for both the sucrose solutions and beverages, while two other clusters had sweetness optima that were at increasingly lower sucrose concentrations. Participants were also asked to rate their liking for common sweet and savoury foods, presented as a list, with Cluster 1 giving higher scores to a majority of the sweet foods than did the other two clusters. A similar pattern was also evident for the savoury food items. This study also included a taste evaluation of milk and dark chocolate samples. Consistent with the ratings of sweet foods items from the list, cluster 1 gave significantly higher liking ratings for the sweeter milk chocolate. This group Subsequently stronger positive emotions to sweet foods were found in sweet likers than in sweet dislikers, even when liking for those foods did not differ between these two groups [31].

In a large sample study conducted in USA, an association between sweet liker status defined through response to sucrose solutions and beverage intake was found, with sweet likers consuming more energy from all beverages, more sweetened juice and tea, and less water than dislikers and neutrals [32]. In a validation of a forced-choice method of determining sweetness solution optima, associations between these optima and sugar content of favourite cereals and beverages were found [33]. However, in contrast, relatively weak associations between liking for sucrose solutions and selected sweet foods were found in two large samples in Finland and Great Britain [34].

Since food preferences will depend upon a range of sensory and non-sensory factors other than their primary tastes, it is difficult to assess the relationships between responses to those tastes and preferences for real foods. One approach is to manipulate the tastant concentrations within foods or beverages and correlate these with hedonic responses to solutions. Hence, Kim et al. [18] used both solutions and simple sweet beverages to assess sweet liker groups. Patterns of responses over the different sucrose concentrations were generally aligned for two of the groups, while the middle group (moderate sweet likers) had optima for the beverages that higher than for solutions, especially at the higher sucrose concentrations. In a comparison of sweet solution-defined sweet likers and dislikers of the concentration at which added sugar in orange juice produced a decrease in liking, this “rejection threshold” was only found for sweet dislikers [35].

Several studies have investigated the relationship between sweet liking status and intensity of sweetness, and their results have been mixed. The majority of the studies did not find any differences in sucrose-induced sweet taste perception by sweet liker status [18,26,32,36,37,38,39,40,41,42], while a few studies reported higher overall sweetness intensity in sweet dislikers than in the other phenotypes [20,43,44,45,46]. Approaches have been developed to link sensory to hedonic data [47,48] and to identify sensory levels at which liking is optimal [49], but in general the role of individual differences in sensory responses has been underexplored. Furthermore, it is well known that sweetness interacts with the perception of other tastes in a mixture (e.g., [10,50,51]).

Other than sensory factors, many variables pertaining to the individual are known to play a role in the preference for sweet and bitter foods, such as gender, age, and personality traits. Liking for sweet foods decreases with age [52] even if was found to be higher again in the elderly [53]. Furthermore, it seems to be higher in men [53], but other studies report a higher liking for sweet foods in women, with an interaction between age and gender, with men over 50 years of age who showed heightened liking for processed sweet foods [34].

Liking of pungent and spicy foods and intake of chilis or spicy food was found to be positively correlated with sensitivity to reward [54,55,56], while individuals higher in food neophobia [57], sensitivity to punishment [58] or sensitivity to disgust [59] reported significantly lower preferences and consumption of vegetables that were characterized by bitterness and astringency and lower preference for coffee/tea without sugar [30]. In addition, food neophobia, sensitivity to disgust and alexithymia (inability of individuals to identify and name their emotional states [60]) were associated with a lower consumption of coffee/tea without sugar [30]. Higher levels of alexithymia were also found to be associated with higher liking of alcohol, sweets, and fats/meats [61]. Furthermore, a recent study showed that personality traits are also associated with differential sensory responses to pungent foods, with higher neophobia, sensitivity to disgust (in both genders), sensitivity to punishment and alexithymia (in women) that reported greater overall pungency intensity [56].

In this explorative study, we aimed at identifying groups of consumers differing in the relationship between their sensory perception of semi-solid samples and their liking for these samples. To this end, increasing concentrations of sucrose were added in a chocolate pudding base. Therefore, the objectives of this study are threefold: (1) to investigate if individual differences in the sensory responses to sweetness, bitterness and astringency are associated with different sweet liking patterns (optima) derived using a product context, (2) to define the psychological and oro-sensory profile of sweet liker phenotypes in a product context, and (3) to assess if individuals differing in sweet liking patterns (optima) differ also in consumption and liking of phenol-rich foods and beverages as a function of their sensory properties (e.g., sweeter vs. more bitter and astringent products). The main idea behind this study is that sweetness optima are associated with different sensory responses in a product context and to different psychological and oro-sensory individual profiles.

## 2. Materials and Methods

### 2.1. Participants

The participants (*n* = 1208; 58.36% women; 41.64% men) had a mean age of 35.5 years (Standard Deviation, SD 12.9; total age range: 18–69 years; age class distribution: 18–30: 45.46%, 31–45: 27.89%, 46–69: 26.65%; age class distribution by gender: 18–30: 59.53% women, 40.47% men; 31–45: 56.51% women, 43.49% men; 46–69: 58.51% women, 41.49% men). Data were collected in eight cities in different geographical areas of Italy (North: Trento, Milan, Pollenzo (Cuneo); Centre: Bologna, Florence; South: Napoli, Potenza, and Catania). Exclusion criteria were pregnancy and breastfeeding at the time of testing, and not being born in Italy or lived there for less than 20 years. Participants (untrained subjects) were recruited by means of announcements, social networks, participant universities and research centres’ websites, national newspapers, and magazines.

All testing involving human subjects was in compliance with the principles laid down in the Declaration of Helsinki, and all subjects provided informed, written consent prior to participation in agreement with the Italian ethical requirements on research activities and personal data protection (Law Decree 30.6.03, 196). The protocol of the studies was approved by the Ethics Committee of the University of Florence.

### 2.2. Overview of Data Collection

**Figure 1:**
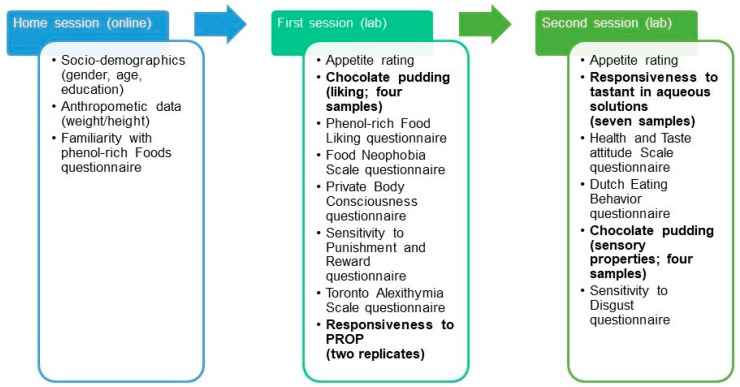
Overview of data collections. Sensory tests that included the evaluation of samples (food products or solutions) are emboldened.

Participants were asked to fill in an online questionnaire before attending two sessions at the laboratory. This included socio-demographic information (self-reported gender, age, education), anthropometric data including weight (kg) and height (m) for the body mass index (BMI) calculation (in kg/m^2^), amount of sugar usually added to coffee (numbers of teaspoons) and familiarity with phenol-rich foods. In the first lab session, participants initially rated their liking for four samples of chocolate pudding spiked with sucrose. Then they completed the Food Liking Questionnaire, the Food Neophobia Scale, Private Body Consciousness, Sensitivity to Punishment and Reward and Alexithymia questionnaires, and at the end of the session they rated the intensity of PROP solutions. In the second lab session, participants rated basic tastes, astringency and pungency in water solutions and then filled in the Health and Taste Attitudes Scale and the Dutch Eating Behavior Questionnaire. Afterwards they evaluated the intensity of sensory properties (sweetness; bitterness; astringency and overall flavour) of the chocolate pudding series and they completed the Sensitivity to Disgust questionnaire. Before each test, participants received detailed instructions and were familiarised with the target sensations (basic tastes, astringency, and pungency) and trained in the use of the scales (Figure 1).

### 2.3. Procedure

#### 2.3.1. Sensory Tests

To control for alliesthesia [62], participants were asked to rate their appetite using a 0–100 visual analog scale (“How hungry are you?; range: “Not at all/Very much”) and then were presented with a series of four samples of chocolate pudding, each spiked with sucrose at different concentrations. The samples were prepared the day before the test by dissolving in water (1:1) an instant chocolate pudding mix (98 g/kg “prepared for pudding to be sweetened, chocolate flavour”, Cameo S.p.A., Brescia, Italy) and bitter cocoa powder (40 g/kg Perugina, Nestlè, Italy) and sucrose at different concentrations (CHOC1, 38; CHOC2, 83; CHOC3, 119; CHOC4 233 g/kg). The samples were cooked in the microwave (900 W) for six minutes and then for 4 min (450 W), mixing every 2 min, then stored in the fridge (4 °C) and removed 2 h before the test to be served at room temperature. Concentrations were selected to elicit a variation in the strength of sweetness (from weak to strong) based on preliminary studies [63]. Cocoa/chocolate was selected for its high content in phenols and its bitter taste and astringent. Participants were asked to rate their liking for these samples using the Labelled Affective Magnitude scale (LAM; [64]) on day 1 and the intensity of the samples’ sensory properties (sweetness, bitterness, astringency and overall flavour) using the general Labelled Magnitude Scale (gLMS; [65]) on day 2. The evaluation of liking and sensory properties was separated in different sessions to avoid any interaction between the two types of evaluation [66]. The presentation order of the samples was randomized across subjects. During tasting, subjects were presented with the samples (15 g) served in small covered plastic glasses, instructed to take a full spoon of chocolate pudding, wait for 10 s, then swallow and evaluate the intensity of the sensations after 3 s. The order of attribute evaluation was randomized, with the exception of overall flavour that was always evaluated last.

Participants rated the intensity of seven water solutions, corresponding to five basic tastes, astringent and pungent sensations, using the gLMS. The concentration of the tastants were decided based on published psychophysical data [63] in order to select solutions equivalent to moderate/strong on a gLMS (sourness: citric acid 4 g/kg; bitterness: caffeine 3 g/kg; sweetness: sucrose 200 g/kg; saltiness: sodium chloride 15 g/kg; umami: monosodium glutamic acid salt 10 g/kg; astringency: potassium aluminium sulphate 0.8 g/kg; pungency: capsaicin 1.5 mg/kg). During tasting, participants were instructed to hold the whole water solution sample in their mouth for 3 s, then expectorate, wait 3 s (5 s in the case of bitterness, umami, astringency and pungency) and evaluate the intensity of the target sensation. The solutions were presented in a randomised order, with the exception of pungency that was always evaluated last. Before evaluating the following sample (both in the case of chocolate pudding samples and in solutions), participants rinsed their mouth with water (30 s), had a cracker (30 s) then rinsed their mouth again.

Before the evaluation, participants were instructed on the meaning of each sensory property and in the use of the LMS and gLMS scales. For the LMS, the scale anchors were spaced from “greatest imaginable dislike” (0) to “greatest imaginable like” (100), with “neither liked nor disliked” set at 50 [67]. Numerical labels were not reported on the scale. For gLMS, the bottom of the scale reflected “no sensation” while the top of the scale represented “the strongest imaginable sensation of any kind” that the participants could imagine experiencing. Participants were asked to recall a variety of sensations from different independent modalities including loudness, oral pain/irritation to provide examples [68]. In order to practice the use of the scale, participants rated intensities of the brightest light they had ever seen, following the procedure described in Dinnella et al. [14].

#### 2.3.2. PROP (6-N-Propyl-2-Thiouracil) Responsiveness

A 3.2 mM solution of 6-n-propyl-2-thiouracil (European Pharmacopoeia Reference Standard, Sigma Aldrich, Milano, Italy) was prepared by dissolving 0.5447 g/L in deionized water [11]. Respondents evaluated the bitter intensity of two identical samples (10 mL) using the gLMS, presented monadically in white plastic cups and coded with two different three-digit codes [7]. Participants were instructed to hold each sample in their mouth for 10 s, expectorate, and then wait 20 s before evaluating the bitterness intensity. In order to control for carry-over effects after the first evaluation, a break of 90 s was taken between the two evaluations. During this break, participants rinsed their mouth with water (30 s), had a cracker (30 s) then rinsed their mouth again. Participants were grouped according to their PROP status on the basis of arbitrary cut-offs reported in previous studies [63,69,70] corresponding to Non Taster (NT; gLMS ≤moderate, 17), Medium Taster (MT) and Super Taster (ST; gLMS ≥very strong, 53).

#### 2.3.3. Personality Traits and Attitudes towards Food

##### Personality Traits

*Toronto Alexithymia Scale (TAS-20).* Alexithymia is a construct characterised by the difficulty identifying subjective emotional feelings and distinguishing between feelings and the bodily sensations of emotional arousal, difficulty describing feelings to other people, an impoverished fantasy life, and a stimulus-bound, externally oriented cognitive style. The trait was measured using the TAS-20 questionnaire, structured in three domains: DIF, difficulty identifying feeling; DDF, difficulty describing feeling; EOT, externally oriented thinking. The questionnaire included a total of 20 items evaluated on 7-point Likert Scales (anchors: 1 = disagree strongly, 7 = agree strongly), [60] validated in Italian by Bressi et al. [71]. The individual score for each domain corresponded to the sum of ratings (ranging from 20 to 100), with higher scores indicating greater level of alexithymia reflected in lower capabilities of identifying feelings (DIF, range 7–35), describing feelings (DDF, range 5–25) and external oriented thinking (EOT, range 8–40).

*Private Body Consciousness (PBC).* The disposition to focus on internal bodily sensations (awareness of internal sensations) was quantified using the 5-items PBC questionnaire [72], validated in Italian by Spinelli et al. [56], on a 5-point scale ranging from 1 = extremely uncharacteristic to 5 = extremely characteristic. The final individual score for PBC is the sum of the scores (range: 5–25).

*Sensitivity to Punishment and Sensitivity to Reward (SPSRQ).* The SPSRQ was used to evaluate the responsiveness of the two brain systems that control the Behavioural Activation System (BAS) and the Behavioural Inhibition System (BIS) [58] validated in Italian by Spinelli et al. [56]. The questionnaire included two scales: the sensitivity to punishment scale (SP) which was built with items that reflect individual differences in reaction and responsiveness to BIS, while the sensitivity to reward scale (SR) was represented by items that measure the BAS functionality dealing with specific rewards (i.e., money, praising, social power). Each scale was rated with a yes/no format and the total score for each subject is represented by the sum of “yes” answers. Based on Spinelli et al. [56], we removed 7 items from the Italian version (4,8,16,25,32,24,26); thus, the scores range from 0 to 23 for SP and from 0 to 18 in SR, with higher scores reflecting, respectively, higher sensitivity to punishment and to reward.

*Food Neophobia Scale (FNS).* Food neophobia describes the reluctance to eat and try new and unfamiliar products. The scale, developed by Pliner and Hobden [57] validated in Italian by Laureati et al. [73], contains 10 items, each evaluated on a 7-point Likert scale (range: 1 = disagree strongly; 7 = agree strongly). The final score for FNS was represented by the sum of ratings (ranging from 10 to 70), after reverse scoring when appropriate.

*Sensitivity to Disgust (DS-SF).* The individual sensitivity to core disgust was evaluated with the 8-item (two subscales) DS-SF questionnaire [74], a short form of the Disgust Scale [75,76] modified by Olatunji et al. [77], validated in Italian by Spinelli et al. [56]. A 5-point scale was used and, in the first subscale, scores rated from 1 (strongly disagree/very untrue about me) to 5 (strongly agree/very true about me), whilerates for the second subscale ranged from 1 (not at all disgusting) to 5 (extremely disgusting). The total score for sensitivity to disgust is given by the sum of scores (ranging from 8 to 40), after reverse scoring when appropriate.

##### Attitudes toward Foods

*Dutch Eating Behavior Questionnaire (DEBQ).* The Dutch Eating Behavior Questionnaire was used to measure individual differences in emotional eating (in response to internal emotional factors), external eating (in response to external factors such as the sight and smell of food), and restrained eating (eating less than desired to lose or maintain a particular body weight) [78], validated in Italian by Dakanalis et al. [79]. The DEBQ consists of 33 items rated on a 5-point scales (1 = never; 2 = seldom; 3 = sometimes; 4 = often, and 5 = very often). The total score for each domain was calculated as the mean, after reverse scoring when appropriate.

*Health and Taste Attitudes Scale (HTAS).* The individual disposition towards the hedonic characteristics of food was measured with the Health and Taste Attitudes Scale questionnaire [80] validated in Italian by Saba et al. [81]. Only the domains “craving for sweet foods” (CSF) and “food as a reward” (FR) were considered in this study. These two subscales included a total of 12 items, rated on a 7-point Likert scale (anchors: 1 = disagree strongly; 7 = agree strongly). The HTAS for each domain was calculated as the mean of the ratings, after reverse scoring when appropriate.

#### 2.3.4. Familiarity with and Stated Liking for Phenol-Rich Foods

Familiarity with, and stated liking for, phenol-rich foods (presented using words) were measured using a selection of items from the IT-Food Familiarity Questionnaire (IT-FFQ) and the IT-Food Liking Questionnaire (IT-FLQ; [63]), developed within the Italian Taste project to collect information about familiarity with and liking for foods among Italians. The IT-FFQ and IT-FLQ included 17 foods items that referred to vegetables (“chicory”; “rocket and radicchio salad”; “artichoke”; “radish”; “asparagus”; “spinach”; “broccoli”; “cauliflower salad”; “cucumber”; “carrot salad”; “fennel”; “lettuce and valerian salad”; “soybean sprout salad”; “beetroot”; “zucchini”; “chard”; “tomato”), 7 to beverages (“grapefruit juice”; “coffee (without sugar)”; “tea (without sugar)”; “coffee (with sugar)”; “tea (with sugar)”; “lemon iced tea”; “peach iced tea”) and 2 to chocolate (“dark chocolate”; “milk chocolate”), as part of a larger group of 184 items. These foods were selected based on their high phenol content [82]. IT-FFQ items were assessed using a 5-point labelled scale (1 = I do not recognize it; 2 = I recognize it, but I have never tasted it; 3 = I have tasted it, but I do not eat it; 4 = I occasionally eat it; 5 = I regularly eat it; [83]. Stated liking was assessed using a 9-point hedonic labelled scale (anchors: 1 = dislike extremely; 9 = like extremely) [84] with the addition of the option “never tasted it”. The presentation order of the items in both questionnaires was randomised across participants.

For each food category, products differing in sensory properties (sweetness, bitterness, and astringency) were selected, in order to compare the responses for products equal in phenol contents but with different sensory properties (e.g., coffee/tea without and with sugar). Beverages and chocolate were classified as “sweet” or “bitter” based on their content of added sugar, while vegetables were classified as “sweeter”, “more bitter and astringent” or “medium” based on the results of a preliminary study that was conducted using a Check-All-That-Apply (CATA) questionnaire [85] with forced choice (yes/no) (“Which of the following words you consider appropriate to describe the following food products?”). In this preliminary study subjects completed an online CATA questionnaire (surveygizmo.eu) aimed at measuring their sensory perception of vegetables, presented using words (201 subjects: 77.7% females; age range 18–70; mean age 40.3 ± SD 14.1). The list of sensory properties included 19 attributes, 9 of which were considered in this analysis (“sweet”; “bitter”, “astringent”; “mild flavour”, “salty”, “sour”, “flavourful”, “pungent”, and “stinging”).

### 2.4. Data Analysis

Pearson correlation coefficients between liking responses to chocolate pudding samples and, respectively, sweet, bitter, astringent, and overall flavour intensity were calculated for each subject. A k-means clustering analysis on *r* values allowed us to identify three segments (C1-C2-C3), each characterized by a specific sensory-liking pattern and optimum; 70 subjects were not considered in the cluster analysis as it was not possible to calculate their correlation coefficient due to zero variance in the evaluation of at least one sensory property (the participant rated with the same score all the samples); for the vast majority it was the case of astringency, and the scores given were nil or very low (>7). Two-way analysis of variance (ANOVA) models were applied to test the effect of cluster and concentration on liking and sensory responses to samples. Type III Sum of Squares and Least Squares (LS) means were used and Bonferroni post-hoc test was applied (*p* = 0.05).

Cronbach α was calculated for all the personality and attitude questionnaires. One-way ANOVA models were applied to test the effect of cluster on appetite, age, personality traits (FNS, SR, SP, DS, TAS-20 and TAS-20 subscales), PROP responsiveness, attitudes (DEBQ: emotional, restrained, external eating; HTAS: craving for sweet foods, food as a reward), perceived intensity in aqueous solutions, amount of sugar usually added to coffee, BMI and liking for vegetables, beverages and chocolate. The association between cluster and, respectively, gender and PROP status was investigated using chi-square tests.

One-way ANOVA models followed by Bonferroni post hoc test (*p* = 0.05) were applied to test the effect of cluster on liking of phenol-rich foods and beverages, while familiarity with these products was assessed through two-tailed Kruskal–Wallis test followed by multiple pairwise comparisons using the Steel–Dwass–Critchlow–Fligner procedure.

The Cochran’s Q test was applied to check for significant differences among vegetables in the preliminary study and a correspondence analysis was applied to investigate sensory differences among vegetables including only attributes that discriminated significantly between products (*p* < 0.05). Pearson Correlation Coefficients between polyphenol content reported in the Phenol-Explorer database [82] and sensory properties were calculated (*p* = 0.05). Data analysis was performed with XLSTAT v2020.5.1.1040 (Addinsoft, Paris, France).

## 3. Results

### 3.1. Clusters Differing in Sensory-Liking Pattern

Three clusters (C1–C3) were identified based on cluster analysis on correlation coefficients. In cluster 1 (*n* = 443, 38.93%) liking was positively related to sweetness and overall flavour, and negatively to bitterness and astringency; in cluster 2 (*n* = 461, 40.51%) liking was positively related to sweetness, and negatively to bitterness, astringency and overall flavour, while in cluster 3 (*n* = 234, 20.56%) liking was negatively related to sweetness and positively to bitterness, astringency and overall flavour.

**Figure 2:**
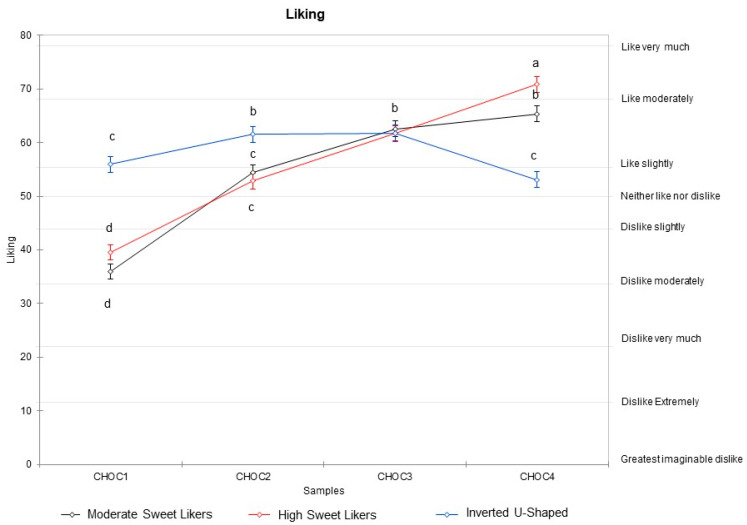
Mean liking ratings for each of the three clusters as a function of sucrose concentration under the four test conditions (CHOC1: sucrose concentration 38 g/kg, CHOC2: 83 g/Kg, CHOC3: 119 g/Kg, CHOC4: 223 g/Kg). ^a,b,c,d^ Different letters indicate a significant difference in the Bonferroni post hoc test (0.05). When the letter is the same for all clusters for the same sample only one letter is reported.

A main effect of sucrose concentration (F_(3, 4540)_ = 493.41, *p* < 0.0001), of cluster (F_(2, 4540)_ = 15.79, *p* < 0.0001) and an interaction between cluster and concentration (F_(6, 4540)_ = 77.2, *p* < 0.0001) on liking were observed. The three clusters differed in their optima and sensory-liking pattern; in cluster 1, liking increased as sweetness intensity increased; in cluster 2, liking increased as concentration increased up to 119 g/kg, at which point it plateaued; in cluster 3, the liking followed an inverted U-shaped pattern with liking increasing up to a sucrose concentration between 83 g/kg and 119 g/kg; and then decreasing to the maximum concentration. Throughout the text, “High Sweet Likers” will refer to cluster 1, “Moderate Sweet Likers” to cluster 2, and “Inverted U-shaped” to cluster 3 (Figure 2 and Table 1).

**Figure 3:**
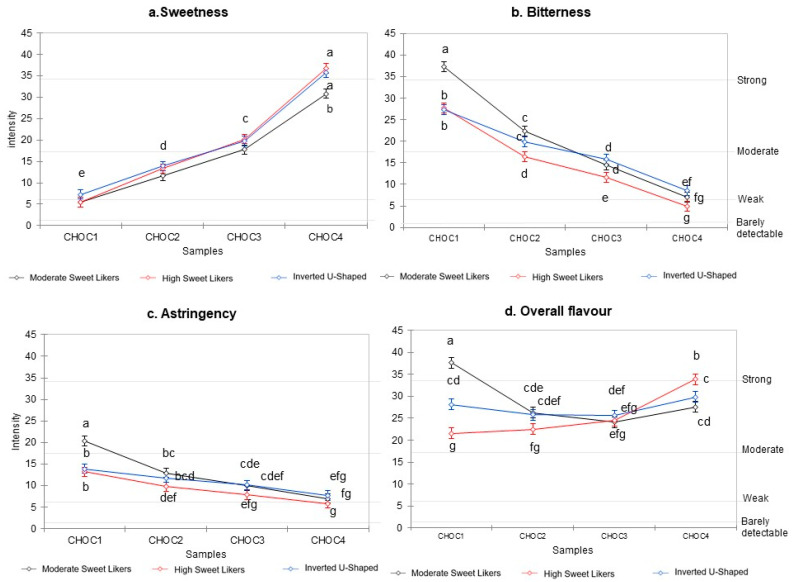
Mean intensity ratings of sweetness (**a**), bitterness (**b**), astringency (**c**) and overall flavour (**d**) for each of the three clusters as a function of sucrose concentration under the four test conditions (CHOC1: sucrose concentration 38 g/kg, CHOC2: 83 g/kg, CHOC3: 119 g/kg, CHOC4: 223 g/kg). ^a,b,c,d,e,f,g^ Different letters indicate a significant difference in the Bonferroni post hoc test (0.05). When the letter is the same for all clusters for the same sample only one letter is reported.

ANOVA models were significant for all sensory properties (Figure 3a–d). The most influential variable was concentration for sweetness, (F_(3, 4540)_ = 1017.43, *p* < 0.0001), bitterness (F_(3, 4540)_ = 654.99, *p* < 0.0001) and astringency (F_(3, 4540)_ = 106.78, *p* < 0.0001). A main effect of cluster, and an interaction between concentration and cluster, were also evident for sweetness (cluster, F_(2, 4540)_ = 24.39, *p* < 0.0001; interaction, F_(6, 4540)_ = 4.96, *p* < 0.0001), bitterness (cluster, F_(2, 4540)_ = 73.06, *p* < 0.0001; interaction, F_(6, 4540)_ = 15.94, *p* < 0.0001) and astringency (cluster, F_(2, 4540)_ = 37.13, *p* < 0.0001; interaction, F_(6, 4540)_ = 8.25, *p* < 0.0001). In the case of overall flavour, the interaction between concentration and cluster had the highest impact on the model (F_(6, 4540)_ = 58.17, *p* < 0.0001), compared to the main effect of concentration (F_(3, 4540)_ = 50.12, *p* < 0.0001) and cluster (F_(2, 4540)_ = 27.96, *p* < 0.0001).

The rated intensity of sweetness, bitterness and, to a lesser extent, astringency followed a similar pattern in the three clusters: increasing, in the case of sweetness, and decreasing in the case of bitterness and astringency, with higher concentrations of sucrose. The three clusters showed limited differences in their evaluation of sweetness in the samples, while differences among clusters were larger in their evaluation of bitterness and astringency, especially for the sample with the lowest concentration of sucrose. The three clusters differed widely in their evaluation of overall flavour, with Moderate and High Sweet Likers judging the sample with the lowest and that with the highest concentration of sucrose, respectively, as the most intense in overall flavour. Conversely, the Inverted U-Shaped cluster reported no change in overall flavour in the four samples.

Compared to the other clusters, the Moderate Sweet Likers rated bitterness and astringency as more intense in the samples with the lowest sucrose concentrations (CHOC1), and the sample with the highest concentration of sucrose (CHOC4) as less sweet. This pattern was not observed for High Sweet Likers who perceived the samples as less bitter and astringent, compared to the other clusters. In fact, High Sweet Likers rated samples as less bitter than Moderate Sweet Likers, and in the case of intermediate concentrations also as less bitter than did the Inverted U-Shaped cluster. They also rated the samples with the lower concentrations in sucrose (38 and 83 g/kg) as less astringent compared to Moderate Sweet Likers, and they perceived the sample with the highest concentration of sucrose as sweeter compared to Moderate Sweet Likers.

No differences in appetite before participating both in the first (liking) and in the second session (sensory responses) was found among the clusters.

### 3.2. Cluster Characteristics

Clusters significantly differed in gender and age (Table 2): a higher percentage of women than expected was found in Moderate Sweet Likers and a lower percentage in High Sweet Likers. The inverted U-Shaped cluster were older than the other clusters.

A main effect of cluster was found for disgust sensitivity, sensitivity to punishment and two TAS-20 (alexithymia) subscales (Table 2). Sensitivity to disgust and was higher in Moderate Sweet Likers than in the other clusters, while sensitivity to punishment and Alexithymia subscale—“Difficulty identifying feelings” were higher in Moderate Sweet Likers, only compared to Inverted U-shaped. Alexithymia subscale—“Difficulty in describing feelings” was higher in Moderate Sweet Likers and in inverted U-Shaped, and lower in High Sweet Likers. There were no significant differences for any of the other personality traits measured.

Clusters differed in PROP responsiveness and in rated intensity of bitterness, umami, and pungency in aqueous solutions, with Moderate Sweet Likers as the most responsive (Table 2). The inverted U-Shaped cluster was the least responsive to PROP, while High Sweet Likers were the least responsive to bitterness, umami and pungency compared to the other clusters. A chi-square test did not show a significant difference between clusters in PROP status distribution, but a trend was observed, with significant chi-square per cell indicating a higher percentage of supertasters in moderate Sweet Likers (chi-square 7.97, *p* = 0.093).

The three clusters did not differ in BMI, nor in attitudes towards foods (HTAS and DEBQ subscales) (*p* > 0.05). On the contrary, large differences were found between clusters in the sugar they usually add to coffee, with the Inverted U-Shaped cluster adding the lowest amount of sugar, compared to the other clusters (Table 2).

### 3.3. Liking and Consumption of Phenol-Rich Food

**Figure 4:**
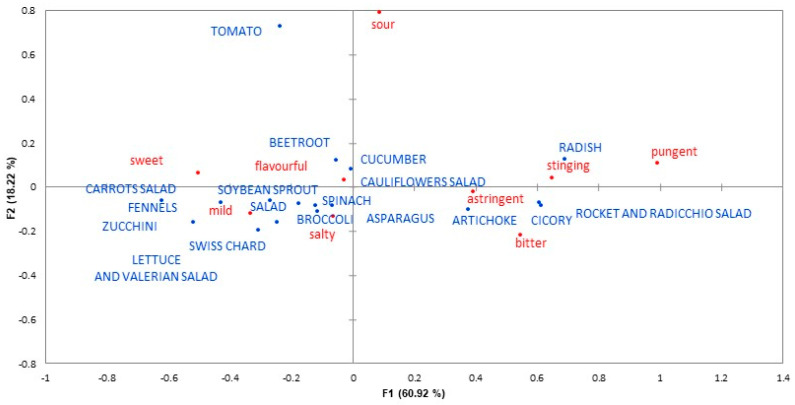
Representation of vegetables and sensory properties based on correspondence analysis after two factors (F1 and F2).

Correspondence analysis in the preliminary study allowed three groups of vegetables to be distinguished by their sensory properties: a group of more bitter/astringent vegetables, a group of sweeter vegetables, and an intermediate group (Figure 4). A significant and moderate to strong correlation was found between astringency and total polyphenol content (*r* = 0.58, *p* = 0.019). No other significant correlations were found between sensory properties and total polyphenol content.

The clusters differed in their liking for, and consumption of, phenol-rich foods (Table 3).

Compared to the other clusters, the Inverted U-Shaped cluster was more familiar with vegetables, particularly those “more bitter”, some of the “medium” and some of the “sweeter”. The differences in liking for vegetables followed the same trend but was found more often for “more bitter” and “medium” vegetables. High Sweet Likers expressed generally an intermediate level of liking compared to the other two clusters, with some exceptions (“rocket and radicchio salad” and “cauliflower salad”), for which they expressed a liking similar to the Inverted U-Shaped cluster. In the case of beverages rich in phenols, when the taste was mitigated by the addition of sugar (e.g., coffee with sugar, sugared iced tea), the Inverted U-Shaped cluster expressed a lower liking and familiarity than the other clusters. Conversely, this cluster showed a higher familiarity and liking for phenol-rich beverages without added sugar, such as coffee, tea and grapefruit juice. Furthermore, the Inverted U-Shaped cluster consumed and liked dark chocolate more than the other clusters. No difference in familiarity with milk chocolate was reported, but High Sweet Likers and Moderate Sweet Likers expressed a higher liking for this product compared to the Inverted U-Shaped cluster.

## 4. Discussion

The approach that we proposed here was applied to a large sample and had the advantage that we were able to consider a response to sweetness in a more ecological context (compared to aqueous solutions) of a semi-solid chocolate product, where sensory properties other than sweetness, such as bitterness, overall flavour and texture, were relevant. Our results suggest that the concentration of sucrose of 119 g/kg (0.35 M) in a chocolate pudding product recorded the highest consensus in acceptability between the clusters, while the Inverted U-Shaped cluster was satisfied also with a lower concentration of 83 g/kg (0.24 M). This latter concentration is in line with the most liked concentration observed in most previous work with Sweet Dislikers using sucrose-solutions [37,86,87] and products [44], although lower values have also been reported [18,21,35].

Our product-based classification of sweet liking is quite similar to that reported using increasing concentration of sucrose in beverages, with similar proportions, albeit here with a higher percentage of Moderate Sweet Likers [18]. In keeping with most of the literature we identified three clusters [86]. However, compared to studies that classified sweet likers based on their response to sucrose aqueous solutions [20,32,88], we did not identify distinct Sweet Dislikers. Instead, in line with Kim et al. [18], we found Moderate Sweet Likers whose sweetness/liking slopes were less steep than those of the High Sweet Likers.

These results confirmed that sweet-liking patterns found with sucrose solutions can be extended to food product contexts, and they point to the existence of a linkage, underestimated so far in the literature on sweet liking based on sucrose solutions, between sensory perception and liking. This study demonstrates that individual differences in sweet liking based on responses to chocolate pudding at different concentrations of sucrose are associated with different sensory responses in a product context. Three main sensory-hedonic patterns were identified: in cluster 1 (“High Sweet Likers”), liking increased as concentration increase, and was positively related to sweetness and overall flavour, and negatively to bitterness and astringency. In cluster 2 (“Moderate Sweet Likers”), liking increased as concentration increased up to 119 g/kg, at which point it plateaued. For these individuals, liking was positively related to sweetness, and negatively to bitterness, astringency, and overall flavour. In the smaller (around 20% of the sample) cluster 3 (“Inverted U-Shaped”), liking increased up to a sucrose concentration between 83 g/kg and 119 g/kg, and then decreased to the maximum concentration. For these participants, liking was negatively related to sweetness and positively to bitterness, astringency, and overall flavour.

It is worth noting that the patterns that we identified here reflect sweetness optima for individuals in the range of concentrations that we selected and could have been alternatively indicated as low, medium, and high sweetness optima in a chocolate pudding matrix. The patterns over different concentrations that we recorded may be a selection from each individual’s inverted-U shape. So, viewed this way, Inverted U-Shaped (Low Sweetness Optimum) whose average optimum is between 83–119 g/kg; Moderate Sweet Likers (Medium Sweetness Optimum) whose optimum is between 119–223 g/kg, and High Sweet Likers (High Sweetness Optimum) whose optimum has yet to be reached. In other words, the identification of specific sweet-liker groups may depend on the range of sweetness values that are being tested, and hence a broader range of concentrations might give a distinctly different range of patterns. Moreover, this would be consistent with what we know about the role of context effects [89] in determining taste intensities and hedonics. Furthermore, it should be considered that also the selected food matrix contributes to context effect.

The current findings also indicate that sensory-hedonic sweet liking patterns are associated with consumption and liking of phenol rich products, such as vegetables and beverages. The inverted U-Shaped cluster reported the highest liking and consumption of vegetables, particularly those characterised by bitterness and astringency, and of beverages without added sugar. In contrast, Moderate Sweet Likers and High Sweet Likers (with some exceptions) consume less vegetables and more sugared beverages compared to the U-Shaped cluster. This points to a higher risk for these latter clusters of unhealthier food behaviours. These results further expand previous findings that showed that individual astringency responsiveness influence the overall acceptability of phenol-rich food items, with more responsive subjects reporting a lower liking [90].

While the greater hedonic differences were found between High Sweet Likers and Inverted U-Shaped, the greatest differences in intensity of the extreme samples (highest or lowest concentration of sucrose) were found between Moderate Sweet Likers and the other two clusters. Instead, High Sweet Likers and Inverted U-shaped had a similar trend in their sensory responses, with the latter showing a slightly lower suppression of bitterness albeit starting from a similar level of perception of bitterness, astringency, and sweetness in the least sweet sample. In contrast, Moderate Sweet Likers differed largely from the other two clusters in sensory response to the samples. This cluster scored the least sweet sample as much more bitter (>strong), astringent (>moderate) and with a higher overall flavour (>strong) compared to the other two clusters. Furthermore, while Moderate Sweet Likers were found to have a greater suppression of bitterness with increasing concentrations of sucrose compared to the other two clusters, this was not reflected in a heightened perception of sweetness in the sweeter samples. In addition, this cluster was found to be more responsive to PROP, compared to Inverted U-Shaped. These results taken together point to a heightened responsiveness of Moderate Sweet Likers to all the sensations, except for sweetness.

In our study, PROP status was associated with differential sensory-hedonic patterns for sweetness in a product context. This differs from previous studies that found no relationship between PROP status and sweet liker status as determined through responses to sucrose aqueous solutions [91,92] or from a finding that supertasters were more likely to be Sweet Dislikers [87]. These discrepancies may be due to the fact that we did not classify sweet likers based on their response to aqueous solutions as in the studies in which PROP ratings have been linked to sweetness intensity [10]. The clusters that we identified, based as they were on sweetness in products, might not correspond to clusters identified through response to aqueous solutions, as suggested by the impacts of taste mixture suppression seen in the current study. In addition, though, preferences for sweetness in products is much more likely to be learnt than is the case with isolated sweet tastes in solution [26].

The largest difference in personality traits between clusters was found in sensitivity to disgust, that was higher in Moderate Sweet Likers compared to the other clusters. Moderate Sweet Likers were also more sensitive to punishment than the Inverted U-shaped cluster, in line with previous studies that reported a positive association between this trait and sugar intake [93]. Clusters differed in two subscales of the alexithymia scale. The values in our population are much lower than the cut-off used to identify clinically alexithymic individuals, but small differences according to sweet liking status were highlighted. Moderate Sweet Likers and High Sweet Likers were higher in the subscale “Identifying feelings”, and Moderate Sweet Likers was also higher in the subscale “describing feelings” compared to High Sweet Likers. This result is partially in line with previous findings that reported that alexithymia was associated to higher liking of sweet [61] and lower of bitter foods [30]. If indeed sweet liker differences are significantly influenced by personality traits, then it is possible that consumption patterns are likewise affected. The core characteristic of alexithymia is marked dysfunction in emotional awareness, social attachment, and interpersonal relation. We may hypothesise that even at low (non-clinical) levels, higher alexithymia may interfere with eating behaviours through an impact on emotional response, as has been recently suggested [94]. It is in fact well known that emotional eating (eating in response to a negative emotion) is associated to higher intake and liking of sweet foods [95].

These differences in personality measures, and particularly in sensitivity to disgust, raise the possibility that the reported difference in terms of sensory optima between Moderate Sweet Likers and the other clusters may have a psychological component in combination with a different sensory response to products, that is heightened in Moderate Sweet Likers. Alternative explanations are possible. One way of thinking about the role of psychological/personality differences in determining sweet liking patterns is to consider that the different hedonic patterns reflect the meaning the individuals in these clusters assign to what they perceive rather than on how intense they perceive it. Indeed, a similar interpretation can be found in our studies of pungency. In these studies, individuals who were more sensitive to disgust expressed both a lower liking for, and a heightened perception of, pungency in foods [56]. Higher levels of disgust sensitivity have also been linked to lower preference for bitter foods [30,96]. Another explanation could be that sensitivity to disgust impacts liking and sensory response to bitter tastes only when in combination with other variables which indicate a heightened individual sensory acuity. However, this second hypothesis seems less corroborated by these data as the differences we reported in PROP responsiveness among clusters are very limited. Anyway, the possible role of age and gender as confounding factors should be considered, as it is well known that these variables may account for different individual sensory acuity levels, with women that are more likely to be supertasters and a decline in taste perception with age [14,97]. We found a higher percentage of women than expected in the Moderate Sweet Liker cluster and a lower percentage in the High Sweet Liker cluster. This suggests that, on average, men have an optimal level of sweetness that is higher than women, which is consistent with previous findings that report of slightly higher liking for sweet foods and beverages in men compared to women [98]. The individuals in the Inverted U-shaped cluster were older on average than the other clusters, in contrast to reports of no age differences [32] or of increases in sweet liking with age in men [34]. However, our results are consistent with previous findings of a decline with age of the heightened sweet preferences in children or adolescents compared with adult populations [52,99,100,101].

The clusters did not differ in craving for sweet foods, that has been previously associated with sweet liking [34], and sensitivity to reward measured using the SPSRQ [58], or in the attitude of using food as a reward, measured through the subscale of the HTAS [80]. Previous studies have reported an association between sensitivity to reward and some unhealthier behaviours, such as higher fat intake, higher alcohol consumption, smoking frequency, while only a trend was observed with sugar intake [93,102]. Furthermore, no difference between the clusters was found in BMI, in line with some studies [32] even if the results in the literature are mixed [88]. Nevertheless, the current findings require further investigation to understand more deeply the role of these and other personality traits in food preferences and consumption behaviours, specifically in relation to sweet foods taking into account gender differences.

## 5. Conclusions

This study suggests that it is possible to identify clusters of subjects based on their sensory-hedonic patterns in a product context. The approach proposed extends the understanding of the liking for sweet taste considering its associations with individual sensory responses to a chocolate pudding presented with different levels of sweetness. This study allowed us to identify three sweet liker phenotypes and has showed that while for one cluster, Moderate Sweet Likers, the liking pattern is related to a higher responsiveness to warning sensation (bitterness and astringency) and a strong suppression of bitterness with increasing concentration of sucrose not associated with a large increase in sweetness perception, this is not the case of High Sweet Likers. A third phenotype was identified, Inverted-U Shaped cluster, that has a lower optimum of sweetness compared to the other clusters and also a lower suppression of bitterness. Differences between clusters were found in terms of age, gender, and personality. These findings point out the importance of identifying individual sensory-liking patterns in order to develop more effective strategies to promote the acceptability of healthy phenol-rich foods. The Inverted-U Shaped cluster was found to have overall healthier food behaviours and preferences, with higher liking and consumption of phenol-rich vegetables and beverages without added sugar. On the contrary, bitterness and astringency might be perceived as barriers for the acceptability and consumption of vegetables and non-sugared beverages by the Moderate and High Sweet Likers.

## Figures and Tables

**Table 1:** Class centroid for each cluster.

Cluster	Sweet	Bitter	Astringent	Overall Flavour
C1 High Sweet Likers	0.765	−0.662	−0.373	0.567
C2 Moderate Sweet Likers	0.582	−0.713	−0.488	−0.580
C3 Inverted U-Shaped	−0.194	0.327	0.309	0.118

**Table 2:** Cluster characteristics expressed in percentages (%) or mean values. Only variables that discriminated significantly among clusters were reported (*p* < 0.05).

Variables	High Sweet Likers	Moderate Sweet Likers	Inverted U-Shaped	Chi-Square/F	*p*-Value
Gender (%)					
women	35.15% <	43.33% >	21.52 %	9.54 (Chi-square)	0.008
men	44.14 % >	36.61% <	19.25 %		
Age (mean)	35.53 ^b^	34.22 ^b^	38.29 ^a^	7.84 (F)	0.0004
Age class (%)					
18–30	44.44	50.44 >	37.04 <	17.93 (Chi-square)	0.001
31–45	31.52	25.33	27.98		
46–69	24.04	24.23	34.98		
Personality Measures					
Sensitivity to disgust	28.85 ^b^	30.05 ^a^	28.55 ^b^	7.58 (F)	0.001
Sensitivity to punishment	9.17 ^ab^	9.93 ^a^	8.79 ^b^	4.38 (F)	0.013
TAS-20—Difficulty Identifying Feeling	15.57 ^ab^	15.60 ^a^	14.63 ^b^	3.72 (F)	0.024
TAS-20—Difficulty describing Feeling	12.46 ^b^	13.26 ^a^	13.49 ^a^	5.05 (F)	0.007
Solutions					
PROP	42.18 ^ab^	44.91 ^a^	38.63 ^b^	4.22 (F)	0.015
Bitter *	29.84 ^b^	34.40 ^a^	31.50 ^ab^	5.72 (F)	0.003
Umami *	26.08 ^b^	29.40 ^a^	27.84 ^ab^	3.52 (F)	0.03
Pungent *	44.84 ^b^	49.84 ^a^	45.52 ^ab^	6.06 (F)	0.002
Usual amount of added sugar in coffee (in teaspoons)	0.83 ^a^	0.77 ^a^	0.56 ^b^	11.53 ** (F)	<0.0001

* in aqueous solution. ** Only individuals that declared that they consumed coffee were included in the analysis (*n* = 956); < and > indicate that the observed value is significantly lower or higher than the expected theoretical value (chi-square per cell significant for ά = 0.05; Fisher exact probability test). ^a,b^ Different letters indicate a significant difference in the Bonferroni post hoc test (0.05). PROP, 6-n-propylthiouracil; TAS-20: Toronto Alexithymia Scale.

**Table 3:** Familiarity and liking of phenol-rich foods differing in sensory properties (products and product class) by cluster.

Sensory Product Class	Foods and Beverages	Familiarity				Liking				
		High Sweet Likers	Moderate Sweet Likers	Inverted U-Shaped	*p*-Value	High Sweet Likers	Moderate Sweet Likers	Inverted U-Shaped	*p*-Value	Never Tasted (%)
Chocolate										
Bitter	Dark chocolate	**562.91** **^b^**	**541.11** **^b^**	**623.39** **^a^**	**0.003**	**7.16** **^b^**	**6.83** **^b^**	**7.92** **^a^**	**<0.0001**	0.35
Sweet	Milk chocolate	582.69	570.48	528.13	0.072	**7.06** **^a^**	**6.75** **^ab^**	**6.47** **^b^**	**0.001**	0.62
Vegetables										
More bitter	Chicory	**560.38** **^b^**	**543.66** **^b^**	**623.12** **^a^**	**0.006**	**6.04** **^b^**	**5.87** **^b^**	**6.53** **^a^**	**0.001**	9.05
	Rocket and Radicchio salad	**574.63** **^a^**	**526.23** **^b^**	**630.70** **^a^**	**0.000**	**6.67** **^a^**	**6.13** **^b^**	**6.81** **^a^**	**<0.0001**	4.39
	Artichoke	**558.47 ^ab^**	**551.76 ^b^**	**610.72 ^a^**	**0.039**	**7.13** **^ab^**	**6.91** **^b^**	**7.49** **^a^**	**0.003**	1.14
	Radish	**556.92** **^b^**	**545.26** **^b^**	**626.49** **^a^**	**0.003**	5.53	5.34	5.76	0.066	9.75
Medium	Asparagus	**547.67** **^b^**	**555.08** **^b^**	**624.53** **^a^**	**0.003**	**7.05** **^ab^**	**6.85** **^b^**	**7.29** **^a^**	**0.023**	1.58
	Spinach	**541.50** **^b^**	**574.48** **^ab^**	**597.85** **^a^**	**0.040**	7.23	7.30	7.35	0.684	0.35
	Broccoli	568.68	549.22	596.50	0.137	**6.95** **^ab^**	**6.64** **^b^**	**7.14** **^a^**	**0.008**	0.79
	Cauliflower salad	**574.76** **^ab^**	**529.86** **^b^**	**623.28** **^a^**	**0.001**	**6.30** **^a^**	**5.86** **^b^**	**6.65** **^a^**	**<0.0001**	5.01
	Cucumber	550.42	567	595.81	0.196	5.60	5.70	6.05	0.085	0.97
Sweeter/ less bitter	Carrot salad	548.81	573.68	585.66	0.244	7.12	7.00	7.23	0.193	0.97
	Fennel	**542.94** **^b^**	**564.44** **^ab^**	**614.95** **^a^**	**0.011**	**6.79** **^b^**	**6.79** **^b^**	**7.20** **^a^**	**0.019**	0.53
	Lettuce and Valerian salad	557.17	558.11	600.64	0.134	7.23	7.12	7.38	0.156	5.98
	Soybean sprout salad	**550.40** **^b^**	**552.03** **^b^**	**625.40** **^a^**	**0.005**	5.62	5.42	5.51	0.457	33.48
	Beetroot	**568.59** **^b^**	**531.36** **^b^**	**631.94** **^a^**	**0.000**	5.45	5.25	5.70	0.089	16.34
	Zucchini	550.43	567.38	595.03	0.092	7.57	7.65	7.73	0.413	0.44
	Chard	559.94	553.45	604.62	0.103	6.24	6.34	6.63	0.093	7.21
	Tomato	560.65	567.87	574.81	0.722	7.83	7.77	7.84	0.798	0.44
Beverages										
Bitter	Grapefruit juice	**573.99** **^ab^**	**536.68** **^b^**	**611.26** **^a^**	**0.006**	**5.47** **^a^**	**5.07** **^b^**	**5.75** **^a^**	**0.0005**	2.55
	Coffee (without sugar)	**541.77** **^b^**	**546.81** **^b^**	**651.97** **^a^**	**<0.0001**	**4.54** **^b^**	**4.55** **^b^**	**5.93** **^a^**	**<0.0001**	2.99
	Tea (without sugar)	**529.33** **^b^**	**565.94** **^b^**	**637.65** **^a^**	**0.000**	**5.00** **^b^**	**5.29** **^b^**	**6.16** **^a^**	**<0.0001**	2.72
Sweet	Coffee (with sugar)	**596.85** **^a^**	**563.27** **^ab^**	**515.70** **^b^**	**0.005**	**6.32** **^a^**	**5.91** **^a^**	**5.34** **^b^**	**<0.0001**	1.76
	Tea (with sugar)	584.27	565.85	534.31	0.131	**6.33** **^a^**	**6.05** **^a^**	**5.60** **^b^**	**0.0002**	0.62
	Lemon iced tea	**585.85** **^a^**	**576.40** **^a^**	**510.50** **^b^**	**0.005**	**6.60** **^a^**	**6.42** **^ab^**	**6.11** **^b^**	**0.013**	0.62
	Peach iced tea	**583.26** **^a^**	**581.63** **^a^**	**505.05** **^b^**	**0.002**	**6.23** **^a^**	**5.95** **^a^**	**5.48** **^b^**	**0.0001**	0.88

*p*-values, mean of ranks for Kruskal–Wallis test (familiarity), mean values for the analysis of variance (ANOVA) models (liking), and % of people who declared to have never tasted the product and thus did not responded to the liking questionnaire are reported. Significant differences are emboldened. ^a,b^ Different letters indicate a significant difference in the Bonferroni post hoc test (0.05).

## Data Availability

The data presented in this study are available on request from the corresponding author.
